# Snail1 Induced Suppression of Proliferation via EGR1, FOXO1, and CEPBγ Creates a Vulnerability for Targeting Apoptotic and Cellular Senescence Pathways

**DOI:** 10.3390/cancers18030510

**Published:** 2026-02-04

**Authors:** Jack Tran, Samyukta Sundaram, Sukirti Shivpuri, Hunain Khawaja, Cynthia K. Miranti

**Affiliations:** 1 Graduate Interdisciplinary Program in Cancer Biology, University of Arizona, Tucson, AZ 85724, USA; 2 Department of Cellular and Molecular Medicine, University of Arizona, Tucson, AZ 85724, USA

**Keywords:** prostate cancer, metastasis, Snail1 (SNAI1), epithelial mesenchymal transition (EMT), cell cycle, RNA-Seq, ChIP-Seq, p21, EGR1, FOXO1, CEBPγ, apoptosis, senescence

## Abstract

Background/Objectives: The annual ~36,000 prostate cancer (PCa) deaths represent a large clinical unmet need and a call for deeper understanding of PCa metastasis. Epithelial–mesenchymal-transition (EMT) has been used to model metastatic behaviors in numerous cancers including PCa. One hallmark of EMT is cell cycle suppression, but how EMT impacts PCa proliferation remains unclear primarily due to the lack of appropriate models. Methods: We transiently induced Snail1 (SNAI1) expression, an EMT driver expressed in PCa, at physiological levels in three PCa cells lines, C4-2B, 22Rv1, and DU145. We used RNA-seq, ChIP-Seq, bioinformatics, qRT-PCR, shRNA, and immunoblotting to identify mechanisms of Snail1-driven inhibition of proliferation. Results: Snail1 suppressed proliferation and G2/M cell cycle progression, without affecting cell death. Mechanistically, Snail1 upregulated expression of CEBPγ, ERG1, FOXO1, cyclin G1, p21, stress genes SESN3 and SOD3, apoptotic programmers Puma, Bax, and Noxa, and senescence-related laminB1, and downregulated Ki67, cyclins A2 and B2. ChIP-Seq data identified Snail1 direct binding to p21, cyclin B2 and G1, EGR1, and CEPBγ promoters. EGR1 induced FOXO1, and EGR1 was required for Snail1-induced SOD3 and Puma, and suppression of Caspase 3 to prevent apoptosis. The EGR1/FOXO1 axis induced BAX, Noxa, and SESN3. CEBPγ was required for Snail1 induction of Lamin B1 to block Snail1-induced senescence. Conclusions: We identified three new major downstream targets of Snail1 that improve our understanding of the role of EMT in limiting stress signaling, apoptosis, and senescence during cell cycle suppression to create a vulnerability for therapeutic targeting.

## Simple Summary

Metastasis is the primary cause of prostate cancer death. The role of epithelial–mesenchymal-transition (EMT) in prostate cancer metastasis initiation is controversial. Snail1, a known EMT driver, is expressed in primary prostate cancer. To better understand the true physiological impact of EMT on prostate cancer, we inducibly expressed the Snail1 (SNAI1) at physiological levels. We found that Snail1 suppresses cell proliferation through several mechanisms that also protect the cells from apoptosis and senescence. These pathways could explain the induction of dormancy prior to metastatic growth and could create therapeutic vulnerabilities in early stages of metastasis.

## 1. Introduction

Regardless of treatment, the majority of the 36,000 prostate cancer (PCa) deaths, in 2025, were attributed to metastasis [1]. This large unmet need is indicative of gaps in knowledge relevant to PCa metastasis. Epithelial–mesenchymal-transition (EMT) is a paradigm that has been used to model the metastatic behaviors of several cancer types, including breast, pancreatic, and colon cancer [2]. During normal development, embryonic cells execute EMT, allowing epithelial cells to become mesenchymal, to lose their epithelial markers, to stop proliferating, to detach from neighboring cells, and to migrate into destined locations [3]. Snail, Twist, and Zeb are families of EMT transcription factors (TFs) that drive the changes in gene expression that induce these EMT phenoyptes [4]. In the clinical setting, Snail1 (SNAI1) protein is expressed in approximately 68% of tumors [5].

Studies that have investigated the roles of Twist1 and Snail1 in PCa highlight the important fundamental ability of EMT drivers to promote migration/invasion. However, they often fail to increase metastasis in vivo and do not always align with clinical observations [6,7,8,9,10,11]. Most, if not all, PCa studies overexpress EMT drivers under CMV-promoter expression systems, resulting in unreasonably high constitutive levels of EMT drivers [6,12]. In some cases, exogenous Snail1 manipulation is more than 200-fold above background levels [12]. Such methodologies cause complete loss of epithelial markers, consistent with classical EMT, but force cancer cells to adaptively bypass EMT-mediated suppression of proliferation, a key aspect of EMT-mediated metastasis.

Elevated Twist1/Snail1 levels predict for poor prognosis for treatment-naïve PCa patients [13]. In the clinical setting, endogenous Snail1 mRNA is elevated 6-fold maximally in PCa samples when AR is suppressed [14] with 68% of the samples expressing Snail1, and yet 86% are expressing high E-cadherin (an epithelial marker) [5]. This co-expression of EMT drivers and epithelial markers in clinical samples has fueled a debate as to the relative importance of EMT in PCa. Studies in breast cancer have suggested a transient induction of EMT is required for initial dissemination, but then reversal of EMT is required for tumor outgrowth at the disseminated site [15,16]. Thus, to truly understand the impact of EMT on PCa metastasis, translational models should recapitulate transient or partial EMT. Strategies that allow for transient/inducible controlled physiological expression of EMT drivers will better highlight natural mechanisms of proliferation suppression.

EMT is increasingly recognized as a dynamic and reversible process rather than a binary developmental switch [15,17]. Recent work has demonstrated that EMT programs can generate a spectrum of intermediate phenotypic states that confer cellular plasticity, enabling cells to reversibly adopt epithelial or mesenchymal traits in response to contextual cues [18,19]. In addition to enabling phenotypic plasticity, EMT-associated programs have been linked to dormancy-like, low-proliferative states that preserve cellular viability and reversibility rather than enforcing irreversible growth arrest or senescence [20,21]. The concept of transient or partial EMT that generates highly motile cells that are non-proliferative, may explain some aspects of tumor latency. Even long after the primary source of cancer cells is removed, the inevitable rise of clinically relevant metastatic tumors implies that disseminated cancer cells experience stages of slow growth and/or metabolism at metastatic sites. For example, after mastectomy, some breast cancer patients will experience a 5-year latent period before developing clinically evident metastatic tumors [22]. A similar observation reported PCa patients with 5–25 years latency [23]. In mouse squamous cell carcinoma models, Twist1 drives metastatic dissemination and suppresses metastatic tumor growth at the same time [15]. Metastatic tumor growth is restored upon Twist1 knockdown, in concert with restored Ki67 expression. Snail1 regulates dissemination and metastatic tumor growth the same way, in breast cancer models [16]. In in vitro PCa models, Snail1 reduces Ki67 expression, and suppression of Snail1 restores Ki67 levels [12]. Understanding mechanisms involving EMT-mediated suppression of proliferation can ultimately identify drivers of metastatic tumor latency.

Cell proliferation and passage through the cell cycle are disrupted in cancer due to oncogenic events associated with constitutive induction of cyclins and activation of cyclin-dependent kinases (CDKs): CDK6/4 and Cyclin D, CDK2 and Cyclin E/A, CDK1 and Cyclin A/B to transient G0, G1, S, G2, M phases of the cell cycle. Oncogenesis simultaneously inactivates cell cycle repressors such as Rb, whose phosphorylation by CDKs is required to inactive it. Meanwhile p21 and p27 act as inhibitors at specific cycle checkpoints, to comply with anti-proliferation signals [24]. Previous studies highlighted inverse relationships between Snail1, cell-cycle progression, Cyclin D/B, Ki67, and p21 [12]. In a canine kidney model, Snail1 bound to the proximal promoter of the Cyclin D1 gene and repressed its expression [20,25]. However, the mechanistic relationships between Snail1 and different steps of the cell cycle have not been identified. Due to the different genetic alterations in cell cycle components that drive oncogenesis, these mechanisms may be different in different cancer types as well as within different subtypes of the same cancer.

Additionally, growth repression in cancer cells can lead to terminal events such as apoptosis or irreversible senescence [22,25]. However, proliferative control is tunable in context-dependent manners [26,27]. Within this framework, transcription factors traditionally associated with stress responses may modulate the proliferative state without enforcing permanent growth arrest (i.e., FOXO1) [20,28]. Understanding how such regulatory programs operate is therefore essential for distinguishing adaptive growth suppression from terminal cell-fate commitment in tumor cells [29].

Structural nuclear scaffolding components have an appreciable impact on proliferative potential. Among these, Lamin B1 (LMNB1) is a key marker of nuclear integrity and proliferative competence [30,31]. Loss of LMNB1 protein is a well-established feature of senescent cells and reflects irreversible nuclear remodeling associated with terminal growth arrest, whereas maintenance of LMNB1 is characteristic of reversible growth restraint [30,32]. Accordingly, changes in LMNB1 abundance provide an important contextual readout for distinguishing adaptive growth restraint from commitment to senescence [32].

In this study, we used an inducible lentiviral expression system to examine Snail1 function at physiologically relevant expression levels in prostate cancer cells. This approach was designed to permit controlled analysis of Snail1-dependent effects on cell-cycle regulation while minimizing artifacts associated with constitutive overexpression. Within this framework, we address how Snail1-associated regulatory programs influence proliferative control without assuming enforced commitment to terminal cell fates, such as apoptosis or irreversible senescence. This strategy enables a more precise evaluation of how Snail1 activity intersects with proliferative regulation in prostate cancer.

## 2. Materials and Methods

Tissue Culture: 22Rv1 [33,34], C4-2B [35,36], and DU145 [37] cell lines were acquired from ATCC. 22Rv1 cells were propagated in RPMI-1640 media, supplemented with 5% FBS, penicillin/streptomycin, and HEPES buffer. C4-2B cells were propagated in DMEM/Ham’s F12 media, supplemented with 5% FBS, Na-Pyruvate, glucose, penicillin/streptomycin, HBSS and HEPES buffers. DU145 cells were propagated in MEM media, supplemented with 5% FBS, non-essential-amino-acids, penicillin/streptomycin, and HEPES buffer. During treatments, 22Rv1 cells were cultured in phenol-red free RPMI-1640, supplemented with 5% charcoal-stripped serum (CSS), penicillin/streptomycin, and HEPES buffer. C4-2B and DU145 cells were cultured in phenol-red free RPMI-1640, supplemented with 2% CSS and HEPES buffer. Treatment media were replaced with fresh media and corresponding drugs every 48 h.

Gene Expression and Knockdown: Snail1 inducible expression was engineered in two plasmid vector systems. For DU145 cells, the lentiviral Tet-ON_*SNAI1*-puro vector was engineered in the VectorBuilder backbone VB230123-1316rfu. For 22Rv1 and C4-2B cells, the Cumate *SNAI1*-puro lentiviral vector was cloned in-house from System Biosciences QM800A back-bone vector. The *SNAI1* cDNA was PCR-amplified from the pLX317-SNAI1 vector [38] (Addgene #115446). The amplified DNA was then cloned, via restriction digestion/ligation via NheI and AsiSI cloning sites, into the QM800A backbone. The lentiviral vector pCCL-cellCycle-BFP-mCherry vector was used for cell-cycle reporter assays [39] (Addgene #132429). To package lentivirus, psPAX2, pMD2.G, and vector of interest were transfected into HEK293T (ATCC). Lentiviral suspension was collected and filtered 48 h post-transfection. Target cells (22Rv1, C4-2B, and DU14) were transduced with collected lentivirus. Target cells were then selected with puromycin to enrich for stable transduction. Snail1 expression was induced with 800 ng/mL of doxycycline or 4.5 µg/mL of cumate. A 2-day pre-treatment of cumate is considered day 0, due to the inherent induction delay. For DU145, there is no pre-treatment. To select for stable transduction of the pCCL-cellCycle-BFP-mCherry reporter, target cells were Flow-sorted with the BectonDickenson FACSAria III instrument, Franklin Lakes, NJ, USA [39]. ForThe pCVM Tag 2B vector carrying Flag-Snail1 was transiently transfected into C4-2B cells, using the PolyPlus JetPrime transfection kit [36]. For iRNA experiments, siControl, siEGR1, CEBPγ, and siFOXO1 pooled-sequences were purchased from Dharmacon, Lafayette, CO, USA (D-001810-10-05, L-006526-00-0005, L-011608-00-0005, and L-003006-00-0005; Table S1). Cells were transfected with 20 nM siRNA, using the PolyPlus JetPrime transfection kit (Fisher Scientific, Waltham, MA, USA) [40]. Cells were treated with vehicle control or cumate/dox to induce Snai1 expression, for 4 d. Cells were then transfected with the siRNA and incubated 2 d more with inducing agent.

Cell Growth/Cell-Cycle Assays: C4-2B cells were seeded at 40,000 cells/cm^2^. Snail1 expression was induced and cells were fixed and stained with crystal-violet for 0, 2, 4, 6, 8 d. Cells were fixed with 4% formaldehyde, washed, and stained with 0.5% crystal-violet in 25% methanol. Cells were rinsed in 25% methanol 5 times. Relative absorbance at 590 nm, using Agilent (Santa Clara, CA, USA) BioTek plate reader was used to quantify staining.

C4-2B and 22Rv1 cells tagged with the cell-cycle reporter pCCL-cellCycle-BFP-mCherry were treated with 4.5 µg/mL cumate to induce Snail1 expression for 6 d. Cells were then fixed with 4% formaldehyde and counterstained with Hoechst (Fisher Scientific, Waltham, MA, USA). Epifluorescence imaging was used to capture mCherry and Hoechst signals. QuPath (v0.5.1) and its AI deep-learning pipelines were used to identify and quantify mCherry-positive cells.

Cell Death Assay: Snail1 was induced in C4-2B cells with 4.5 µg/mL cumate for 6 d. Cells were then assayed for apoptosis per the Cell Signaling Technologies (Danvers, MA, USA) TUNEL kit 25879S [41]. Briefly, cells were fixed with 4% formaldehyde and washed with 1× PBS. Cells were then treated with equilibration buffer and TUNEL reaction buffer for 1 h, sequentially. Cells were washed and counterstained with Hoechst. Epifluorescence microscopy was used to capture FITC/488 positive cells.

Immunoblotting. Treated cells were harvested in RIPA buffer and sonicated to fragment DNA to eliminate viscosity. Protein samples were normalized with 4× Laemmli sample buffer and 1× RIPA buffer to final concentration 1 µg/µL. Samples were denatured at 75 °C for 15 min. A total of 20 µg protein per sample was separated in 10% polyacrylamide gel via electrophoresis. Proteins were transferred into PVDF membrane. PVDF membranes were blocked for 2–4 h in 5% BSA, 0.05% TBS-Tween 20, and 1.25% Goat serum. Primary antibodies were hybridized to PVDF membrane overnight at 4 °C. Secondary antibodies (HRP-conjugated) were hybridized for 1 h at room temperature. Signal was developed by ECL reagent and imaged using the GeneBio Systems (Burlington, ON, Canada) digital acquisition machine. Antibodies and corresponding targets are listed in Table S2. Densitometry: blot signals were quantified through a simple ChatGPT (v5.2) pipeline. For each immunoblot, tight rectangular regions of interest (ROIs) were manually defined around individual signal bands and their corresponding loading control (tubulin) bands. ROIs of identical dimensions were applied uniformly across all lanes for a given target. A background ROI of matching size was used for background subtraction. Densitometry was performed by quantifying all 8-bit grayscale pixel intensities within each ROI, and integrated density was calculated as the summed signal intensity minus the summed background intensity. Target protein abundance was normalized to the corresponding tubulin signal for each lane. Densitometry values shown in figures represent the normalized integrated density multiplied by 100.

qPCR: Extracted mRNA was reverse transcribed into cDNA at 1:1 ratio per QuantBio product catalog# 95048-100. cDNA was then amplified and detected with Invitrogen QuantStudio 3.0 thermocycler (Fisher Scientific, Waltham, MA, USA). Apex Bioresearch Products (Genesee Scientific, El Cajon, CA, USA) A324406 was used as the primary SYBR Green reagent to read out amplified cDNA. Primer sequences were per Table S3. Gene expression was normalized to RPL4 and expressed relative to control.

RNASeq: C4-2B cells stably transduced with the cumate-*SNAI1* lentivirus were pretreated or not with 4.5 µg/mL of cumate for 4 d. Total RNA was extracted and purified using TRIZOL lysis buffer and isopropanol precipitation. Total RNA was submitted to NOVOGENE at 200 ng/µL and polyA mRNA selected for NGS sequencing.

Raw sequencing data was first subjected to quality assessment using the FastQC (v0.11.9) tool to evaluate base quality scores, adapter contamination, and sequence duplication levels. Transcript quantification was performed using Kallisto (v0.48.0), a pseudoalignment-based method that estimates transcript abundance [42]. Reads were aligned against the Ensembl GRCh38 transcriptome index. The output transcript-level abundance estimates were imported into R (v4.4.2) using the tximport package (v1.34.0) to summarize counts at the gene level. Normalization and differential expression analysis were conducted using DESeq2 (v1.46.0). Differentially expressed genes were identified by applying Benjamini–Hochberg correction to adjust *p*-values for multiple comparisons. Genes with an adjusted *p*-value < 0.1 and an absolute log2 fold change > 0.85 were considered significant. Visualizations such as PCA plots, heatmaps, and volcano plots were generated using ggplot2 (v3.5.2). Gene Set Enrichment Analysis (GSEA) was performed using the Broad Institute’s software (v4.3.3) [43] on the full list of ranked genes.

Transcription factor (TF) activity was inferred using the Virtual Inference of Protein-activity by Enriched Regulon analysis (VIPER) algorithm [44], implemented via the viper R package (v1.44.0). DoRothEA regulons [45] were used, consisting of TF–target interactions curated from literature and other sources. Only regulons with confidence levels A–C were used to balance coverage with reliability. Gene expression matrices were z-score normalized before analysis. The resulting normalized enrichment scores (NES) were used to estimate relative TF activity across samples.

ChiP-Seq: 14.5 × 10^6^ C4-2B cells were transiently transfected with mCherry-control or FLAG-*SNAI1*-6SA vector for 2 d (Addgene #16221) [40] using the JetPrime transfection kit Fisher Scientific, Waltham, MA, USA) and protocol (101000015). Cells were harvested per the Cell Signaling Technologies (CST) SimpleCHIP Enzymatic Chromatin IP magnetic kit. Briefly, cells were fixed in 1% EM grade formaldehyde (methanol free) for 10 min at room temperature. Fixation was halted with glycine. Cells were collected with a scraper in ice-cold PBS and protease inhibitors. Cell nuclei were extracted via isotonic lysis and centrifugation. Chromatin was digested/fragmented with micrococcal nuclease for 20 min at 37 °C. Digestion was then halted with EDTA. The digested, but intact nuclei, were then pelleted and resuspended in ChIP buffer. Samples were divided into 100 µL aliquots, in 600 µL microcentrifuge tubes compatible with the Diagenode Biorupter Sonicator (Diagenode, Denville, NJ, USA C3001001-500). Aliquoted tubes were then loaded into the Diagenode Biorupter and sonicated at 30s-ON/30s-OFF for a total of 5 cycles. Sonicated samples were then centrifuged at 9400 RCF, and suspensions were collected for downstream immunoprecipitation. For every sample, 500 µL of digested and sonicated chromatin was hybridized to 1 µg of IgG control, 10 µL of anti-H3 control, or 1.4 µg of anti-FLAG (CST 14793S), as instructed per ChIP kit. Antibodies were hybridized for 4 h at 4 °C. Protein G magnetic beads (Cell Signaling Technologies, Danvers, MA, USA) were used to capture chromatin-antibody complexes. Bead complexes were washed with indicated buffers per kit instructions. Chromatin was eluted, and reverse cross-linking was performed with addition of proteinase K for 2 h at 65 °C. DNA was then purified through standard spin-column elution. Purified DNA was submitted to NOVOGENE (Sacramento, CA, USA) for NGS ChIPseq.

ChIP-seq peak calling and image rendering: Normalized signal intensity tracks (bigwig files) were loaded into the Integrative Genomics Viewer (IGV). Group Auto Scaling was applied across Experimental and Control tracks to ensure that signal heights were proportional to the normalized read density and to prevent the over-magnification of background noise in the Input samples.

To visualize the distribution of Snail1 binding intensity, a signal matrix was generated using deepTools computeMatrix in reference-point mode. Snail1 ChIP-seq and Input BigWig files were centered on the Transcription Start Sites (TSS), spanning a window of 3 kb and a bin size of 10 bp. Heatmap plots were generated using DeepTools (v3.5.6) plotHeatmap. To prevent the artificial inflation of background noise in the Control, both the color scale (zMax) and the profile plot vertical axis (yMax) were fixed to identical values across the Experimental and Input tracks. Heatmap rows were sorted in descending order based on the mean SNAI1 signal intensity.

All computational analyses were performed on the Galaxy web platform (https://usegalaxy.org). Raw sequence quality was evaluated using FastQC (Galaxy Version 0.74+galaxy1). Adapter sequences and low-quality bases (Phred score < 20) were removed using Trimmomatic (Galaxy Version 0.39+galaxy2). Trimmed reads were aligned to the GRCh38 reference genome using BWA MEM (Galaxy Version 0.7.19). Resulting BAM files were sorted and filtered for blacklisted regions using SortSAM (Galaxy Version 3.1.1.0) and bedtools Intersect intervals (Galaxy Version 2.31.1+galaxy0). Peak Calling and Normalization. Identification of enriched genomic regions was performed using MACS2 callpeak (Galaxy Version 2.2.9.1+galaxy0). For each sample, ChIP reads were compared against the input DNA control (C4-2B mCher). The effective genome size was set to H.sapiens 2.7e9, and a q-value (FDR) threshold of 0.05 was applied. For narrow peaks, the standard shifting model was used. To visualize enrichment, signal tracks were generated using MACS2 to produce bedGraph files, and subsequently signal tracks (bigWig) were generated via bamcoverage (Galaxy Version 3.5.4+galaxy0), with normalization to Bin Size 10 and RPKM (Reads Per Kilobase per Million mapped reads) to enable quantitative comparison between samples and visualization in the Integrative Genomics Viewer (v2.19.7). Genomic Annotation and Motif Discovery. Peak annotation relative to the nearest Transcription Start Site (TSS) was performed using the R/Bioconductor package ChIPseeker (v.1.26.0). De novo motif enrichment was calculated using MEME-ChIP (Galaxy Version 4.11.2+galaxy1) using a 100 bp window around peak centers. Statistical significance for motif enrichment was determined by Fisher’s Exact Test, E < 0.05.

Statistical Analysis: Data was graphed and analyzed with Prism Graphpad (v10.2) Error bars were calculated based on Standard Error of the Mean (SEM). Outliers were identified using Graphpad’s outlier calculator, whenever applicable. Student unpaired *t*-test was used to determine statistical significance.

AI Usage: AI was used as part of QuPath (v0.5.1) software for quantification of microscopy images. OpenAI CHAP-GPT v5.1 and v5.2 was used to identify potential pathway interactions from the bioinformatic data. All references cited by AI were verified for correct interpretation.

## 3. Results

### 3.1. Snail1 Suppresses Cell Growth and Cell-Cycle Progression but Does Not Affect Apoptosis

**Figure 1:**
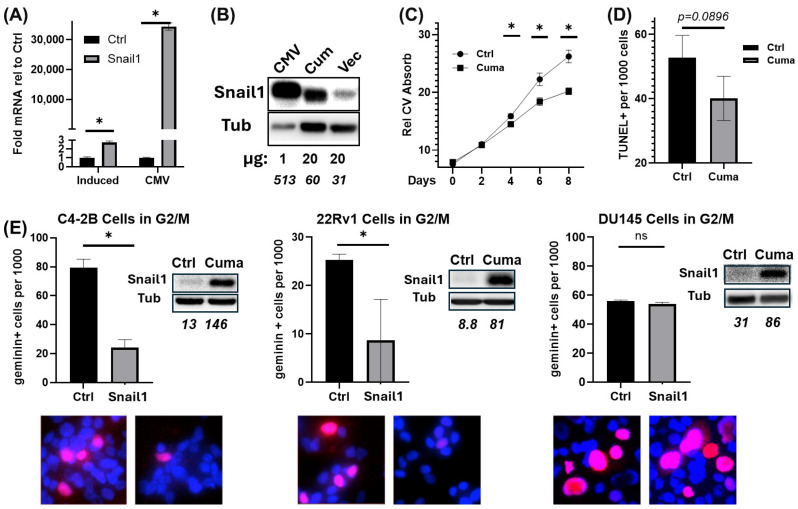
Snail1 suppresses PCa proliferation and cell-cycle progression but does not affect apoptosis. (**A**,**B**) C4-2B cells were either transduced with inducible lentiviral *SNAI1* (Induce) and treated with or without 4.5 μg/mL cumate or transiently transfected with CMV-driven constitutive Snail1 vector or an empty vector. (**A**) Levels of Snail1 mRNA measured by qRT-PCR. (**B**) Levels of Snail1 and tubulin (Tub) assessed by immunoblotting (see Figure S2). (**C**) Snail1 was or was not (Crtl) induced in C4-2B for 0–8 d and stained with crystal violet (CV) every two days. CV stain was quantified on a plate reader. *n* = 4. * *p* < 0.05. (**D**) Snail1 was or was not (Crtl) induced in C4-2B cells for 6 d, fixed and TUNEL stained, imaged with epifluorescence microscopy, and quantified using Qupath. *n =* 3, *p* = ns. (**E**) 22Rv1 and C4-2B stably expressing cumate-inducible Snail1 and Geminin-mCherry were treated or untreated (Crtl) for 6 d with 3 µg/mL and 4.5 µg/mL of cumate respectively, to induce Snail1 expression. DU145 cells were treated with 800 ng/mL doxycycline to induce Snail1 expression for 6 d. Cells were formalin-fixed and counterstained with Hoechst (blue), then imaged using epifluorescence microscopy. mCherry + (red/pink) cells were quantified using QuPath. *n* = 3. * *p* < 0.05. *Inset*: Immunoblotting was used to measure Snail1 induction with tubulin (Tub) as a loading control (see Figure S2). Blot densitometry: bottom row of each blot (italicized numerals).

To determine the functional role of Snail1 in cell proliferation and cell cycle suppression, cumate-inducible lentiviral *SNAI1* was first transduced into C4-2B cells and its induction levels compared to the expression of constitutive CMV-driven Snail1 (Figure 1A,B). Snail1 mRNA was induced ~3-fold in C4-2B cells, but over 34,000-fold under a constitutive CMV promoter. Based on a standard growth curve, inducible Snail1 expression significantly suppressed C4-2B cell growth beginning at day 4 of Snail1 induction (Figure 1C). TUNEL analysis showed that apoptosis did not contribute to the Snail1-induced reduction in cell growth (Figure 1D). After stable transduction of inducible Snail1 into 22Rv1, C4-2B, and DU145 PCa cell lines, they were further tagged with Geminin-mCherry to measure G2/M progression. Upon Snail1 induction, cell-cycle progression in 22Rv1 and C4-2B, but not DU145 cells, was significantly suppressed (Figure 1E). Thus, under physiological levels of Snail1, tumor cell growth and cell cycle progression in the AR+ tumor cells were significantly suppressed in the absence of any impact on cell survival.

### 3.2. Snail1 Induction Leads to Global Inhibition of Cell Cycle Pathways

**Figure 2:**
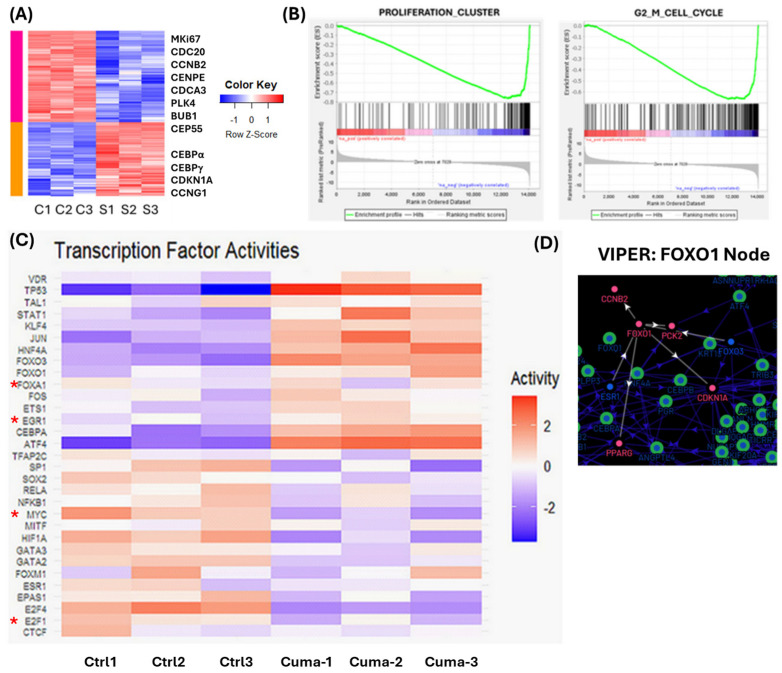
Snail1 induction leads to global inhibition of cell cycle pathways. C4-2B cells were treated with 4.5 µg/mL cumate (Cuma) to induce Snail1 expression or H_2_O vehicle (Crtl) for 4 d. *n* = 3. Total mRNA was extracted and sequenced. (**A**) Heatmap of statistically significant differentially expressed genes from Snail1-induced mRNA (S) versus control (C) in triplicate. Genes strongly associated with cell proliferation and cell cycle were found in differentially expressed genes. (**B**) Top GSEA hits included proliferation and G2/M cell cycle. (**C**) VIPER analysis identified probable transcriptional drivers of differentially expressed genes. (**D**) Pathway node of VIPER predicted FOXO1 regulon showing a relationship to *CCNB2* (Cyclin B2) and *CDKN1A* (p21). White arrows indicate directionality of relationship.

To identify downstream targets of Snail1, we used bulk RNA-sequencing of uninduced versus cumate-induced Snail1 in C4-2B cells for 4 days. Differential gene expression analysis revealed downregulation in cell cycle drivers MKI67, CDC20, CCNB2 (cyclin B2), CENPE, CDCA3, PLK3, BUB1, and CEP55 and upregulation of cell-cycle suppressors CEBPβ, CEBPγ, CDKN1A (p21), and CCNG1 (cyclin G1) (Figure 2A; Table S4). The Snail1-induced mRNA dataset was then run through a GSEA pipeline [46]. The top hits were G2/M cell cycle and cell proliferation (Figure 2B). To identify probable transcriptional drivers behind our RNA-seq dataset, we employed VIPER [45]. By superimposing our dataset onto the human regulon, we identified a set of potential drivers, consistent with observed changes in target genes (Figure 2C). The FOXO1, E2F1, E2F4, CEBPA, MYC, TP53 regulatory nodes are consistent with observed changes in cell growth and upregulation of p21 (CDKN1), cyclins A2 (CCNA2), and B2 (CCNB2). The FOXO1 regulatory node predicts Snail1-induced FOXO1 regulation of p21 and cyclin B2 (Figure 2D).

### 3.3. Snail1 Upregulates ERG1, FOXO1, and p21

**Figure 3:**
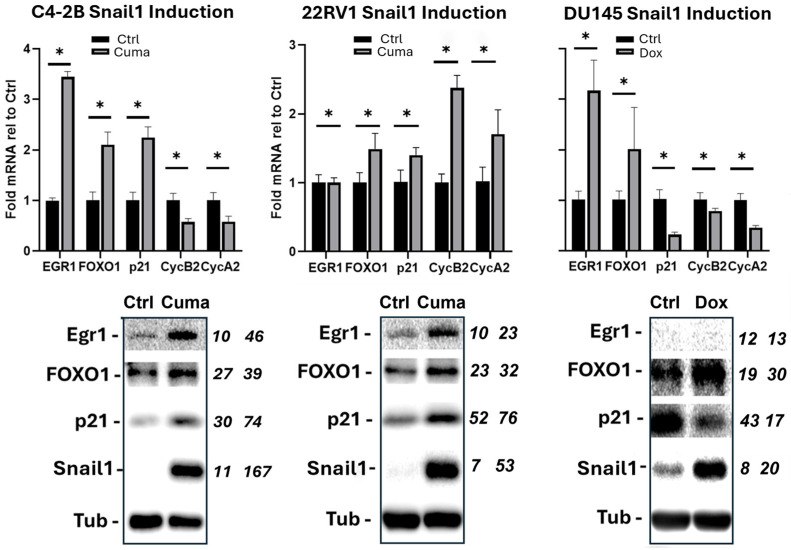
Snail1 upregulates ERG1, FOXO1, and p21. Snail1 was induced in C4-2B and 22Rv1 cells for 6 d with 4.5 µg/mL of 3.0 µg/mL of cumate (Cuma), respectively. DU145 cells were treated with doxycycline (Dox) (800 ng/mL). Protein and mRNA expression measured by qRT-PCR and immunoblotting (see Figure S2). Tubulin served as loading control. Blot densitometry: numerical columns (italicized) to the right of each blot. Right column (veh), left column (Snail1 induced). *n* = 4. * *p* < 0.05.

We validated Snail1 upregulation of EGR1, FOXO1, and p21 mRNA and protein, and downregulated cyclin A2 and B2 mRNA in C4-2B cells (Figure 3). In DU145 cells, Snail1 also controlled EGR1, FOXO1, cyclin A2, and B2 mRNA in the same manner, but failed to induce p21. In fact, p21 mRNA and protein decreased. While EGR1 mRNA increased 3-fold in DU145 cells, there was no detectable protein. These data are consistent with our earlier observation of unchanged cell cycle in DU145 cells with respect to Snail1 induction, suggesting p21 might be a major determinant of proliferation. Consistent with a dominant role for p21, Snail1 upregulated FOXO1 and ERG1 protein and modestly induced p21, whereas cyclin A2 and B2 mRNA were upregulated nearly 2-fold. In spite of this, there was still an impact on cell proliferation in 22Rv1 cells (Figure 1E).

### 3.4. Snail1-Dependent Upregulation of FOXO1 Requires EGR1

**Figure 4:**
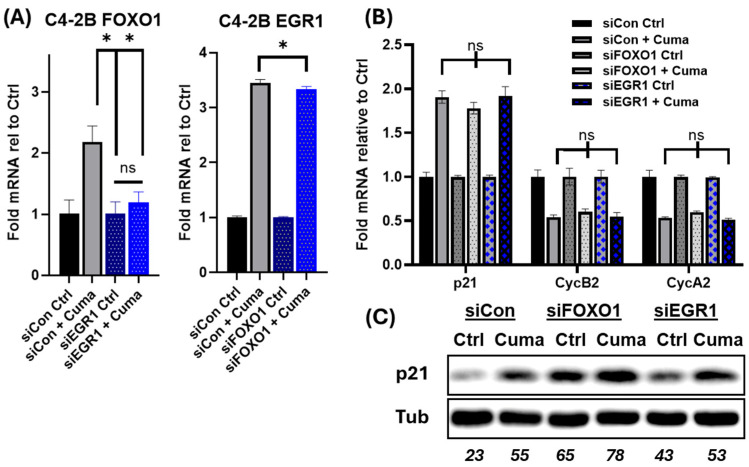
Snail1-dependent upregulation of FOXO1 requires EGR1. (**A**) Snail1 was induced in C4-2B cells for 4 d, then transfected with pooled control siRNA (Con) or sequences to FOXO1 (siFOXO1) or EGR1 (siEGR1) for 2 d. Each knockdown group was normalized to its uninduced control (Ctrl). (**A**) qRT-PCR was used to quantify mRNA for FOXO1 and EGR1 for each knockdown. *n* = 3, * *p* < 0.05. (**B**) Levels of p21, cyclin B2, and cyclin A2 mRNA analyzed by qRT-PCR. (**C**) Total p21 protein analyzed by immunoblotting (see Figure S2). Tubulin (Tub) served as loading control. Blot densitometry: bottom row of blot (italicized numerics).

Mechanistic relationships between Snail1 and FOXO1 have not been previously described. A network visualization of the VIPER analysis suggests that FOXO1 is an upstream regulator of p21 and Cyclin B2 (Figure 2D). Moreover, recent studies on p15 and MMP9 illustrate that EGR1 and FOXO1 directly act together on target promoters to upregulate their expression [47,48,49]. To see if a similar relationship exists for p21, we tested whether ERG1 and FOXO1 are involved in the Snail1-dependent upregulation of p21 or suppression of cyclin A2/B2. We induced Snail1 for a total of 6 days and knocked down either ERG1 or FOXO1 in the last 2 days. Loss of EGR1 prevented Snail1 from upregulating FOXO1 mRNA and protein (Figure 4A), but not vice versus, indicating Snail1 upregulates FOXO1 mRNA and protein at the transcriptional level, through ERG1; which is downstream of Snail1. Although knockdown of FOXO1 did not alter ERG1 mRNA, it did prevent Snail1 from upregulating ERG1 protein, suggesting additional post-transcriptional regulation. However, neither loss of ERG1 nor FOXO1 prevented Snail1 induction of p21 or reversed loss of cyclin A2/B2 (Figure 4B), indicating this is not the primary mechanism for p21 induction by Snail1. Interestingly, loss of FOXO1 upregulated baseline levels of p21 protein (Figure 4C), but not mRNA, suggesting a possible role for FOXO1 in moderating p21 activities overall.

### 3.5. Snail1 Upregulates CEBPγ and p21 Independently

**Figure 5:**
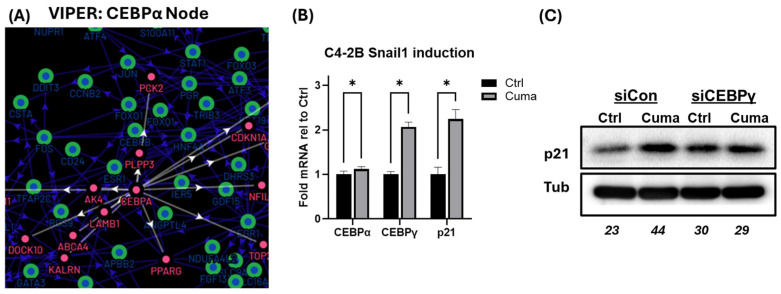
Snail1 upregulates CEBPγ and p21 independently. (**A**) VIPER analysis identified a CEBPα node that controls p21. White arrows indicate directionality of relationship. (**B**) Levels of CEBPα, CEBPγ, and p21 in C4-2B cells induced or non-induced (Ctrl) to express Snail1 measured by qRT-PCR. *n* = 4. * *p* < 0.05 (**C**) Snail1 was induced w/4.5 μg/mL cumate (Cuma) or Control (Ctrl) in C4-2B cells for 6 d. In the last 48 h, cells were treated with siCON or siCEBPγ. Total p21 protein analyzed by immunoblotting (see Figure S2). Tubulin (Tub) served as loading control. Blot densitometry: bottom row of blot (italicized numerals).

VIPER node analysis revealed a probable alternative mechanism of Snail1-dependent p21 upregulation through the CEPB family, particularly CEBPα (Figure 5A). Although the differential-gene-expression in our Snail1 mRNA-seq dataset did not show changes in CEBPα, we observed Snail1-dependent upregulation of CEBPγ and CEBPβ (Figure 2A, Table S4). Lacking precedence of CEBPγ’s role in PCa oncogenesis, compared to CEBPβ, prompted further investigation [50,51,52,53]. qPCR analysis validated CEBPγ upregulation (Figure 5B). We then asked whether CEBPγ plays a role in proliferation in a Snail1-dependent manner. We knocked down CEBPγ during the last 48 h of a 6-day Snail1 induction and probed for p21 and cyclin A2/B2 expression changes. CEBPγ loss did not impact Snail1’s ability to regulate mRNA expression of p21 and cyclin A2/B2 (Figure S1). However, CEBPγ loss resulted in a global increase in the baseline level of p21 protein, with Snail1 unable to further upregulate p21 protein (Figure 5C).

### 3.6. Snail1 Directly Binds to the Promoter Regions of Cell Cycle Drivers, ERG1, and CEBPγ

**Figure 6:**
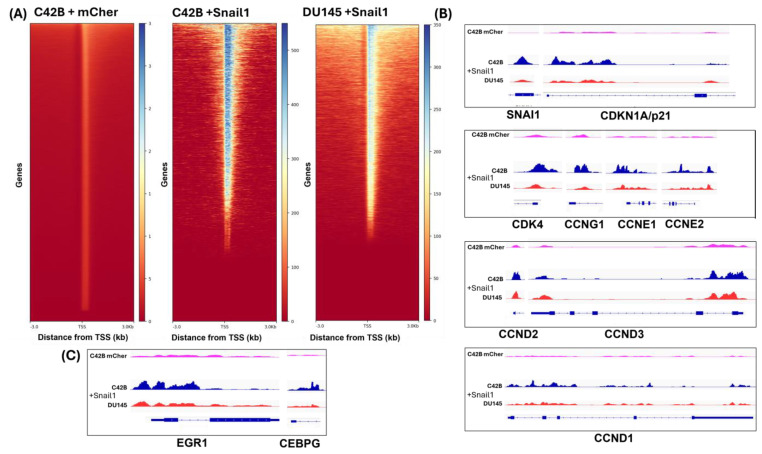
Snail1 directly binds to the promoter regions of cell cycle drivers, *ERG1*, and *CEBPγ*. C4-2B and DU145 cells transiently transfected with negative control mCherry or 6SA-*SNAI1*-FLAG expression vector for 2 d. Anti-FLAG antibodies were used to pull down 6SA-Snail1-Flag via chromatin immunoprecipitation and resulting DNA sequenced. (**A**) Total peaks occupied by Snail1 centric to transcriptional start site (TSS). (**B**) Cell cycle target gene peaks occupied by Snail. (**C**) Snail occupancy peaks on EGF1 and CEBPγ.

Given our previous observations that Snail1 suppresses proliferation, upregulates p21, and that the CEPB node regulates p21 and proliferation drivers like Dock10 and Top2A (Figure 5A), we aimed to clarify why ERG1, FOXO1, or CEBPγ knockdown did not alter Snail1’s ability to regulate mRNA readouts of p21 and Cyclin A2/B2. We performed ChIP-Seq, using anti-FLAG antibodies, against cells transfected with mCherry control, or 6SA-*SNAI*-FLAG expression plasmids [40]. ChIPseq was successful, evident by low peak counts in our negative mCherry control, and high peak counts in our 6SA-*SNAI1*-FLAG for C4-2B and DU145 cells (Figure 6A). Importantly, we showed Snail1 directly binding at the p21 (*CDKN1a*) gene (Figure 6A, Tables S5 and S6). We also showed Snail1 directly binding to the promoters of *CDK4*, cyclins G1, E1, E2, D1 and D3 (*CCNG1*, *E1*, *E2*, *D1*, *D3*) (Figure 6B). Particularly, Snail1 binds directly to the promoter of the cyclin B2 (*CNNB2*), which we showed to be downregulated by Snail1 (Figure 2A and Figure 3). Importantly, we showed that Snail1 directly binds to the *EGR1* and *CEBPγ* promoters (Figure 6C). We did not observe Snail1 occupancy anywhere on the *FOXO1* gene. Snail1 occupancy at the *ERG1* gene, and not *FOXO1*, is consistent with our observed Snail1-EGR1-FOXO1 regulatory axis in Figure 4.

### 3.7. FOXO1/EGR1 Axis Controls Snail1-Induced Stress Responses and CEBPγ Prevents Irreversible Senescence

**Figure 7:**
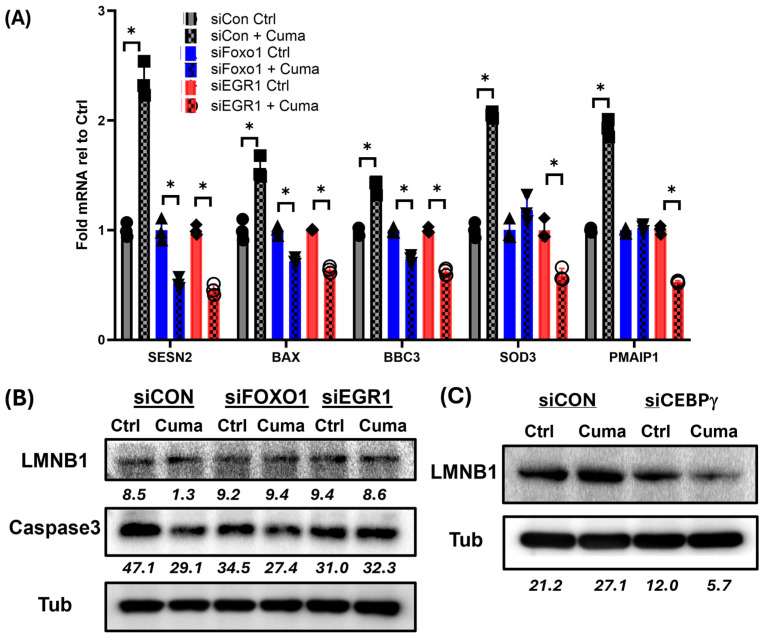
FOXO1/EGR1 Axis controls Snail1-induced stress responses and CEBPγ prevents irreversible senescence. Snail1 was induced in C4-2B cells for 6 d. In the last 48 h, cells were treated with siCON, siFOXO1, siEGR1, or siCEBPγ. (**A**) Fold change in mRNA normalized to control (Ctrl). *n* = 4. * *p* < 0.05. SESN2 and SOD3 are stress-response markers. BAX, BBC3 (PUMA), and PMAIP1 (NOXA) are apoptotic programming markers. (**B**) Immunoblotting for LaminB1 (LMNB1) and total caspase 3 (Casp3) with siCON, siFOXO1, or siEGR1. Tubulin (Tub) serves as loading control (see Figure S2). (**C**) Immunoblotting for LaminB1 (LMNB1) with siCON or siCEBPγ (see Figure S2). Blot densitometry: bottom row of each blot (italicized numerics).

Loss of FOXO1 or CEBPγ resulted in a global baseline increase in p21 protein and limits Snail1 from inducing more p21 protein; despite having minimal impact on how Snail1 induces p21 mRNA (Figure 4C and Figure 5C). Given that p21 has strong links to apoptosis/cell stress and senescence [54,55]. we sought to determine if Snail1 impacts senescence or apoptotic priming through FOXO1 or CEBPγ. Snail1 induced mRNA expression of several stress response/apoptosis priming genes, BAX, BBC3 (PUMA), SOD3 and PMAIP1 (NOXA) (Figure 7A). Knockdown of FOXO1 or EGR1 prevents this induction and further decreases expression (Figure 7A). Interestingly, Snail1 downregulated total caspase3 protein, and this was selectively reversed by EGFR1, but not FOXO1, knockdown (Figure 7B). These results strongly suggest that the absence of apoptosis in Snail1-induced cells is due to EGR1 upregulation to suppress caspase 3 that might be triggered by induction of pro-apoptotic programmers. Lastly, we evaluated the behavior of the canonical nuclear scaffolding senescence marker LMNB1 (laminB1). Snail1 modestly upregulates laminB1, but knockdown of FOXO1 or ERG1 had no impact on laminB1 (Figure 7B). However, knockdown of CEBPγ prevented Snail1 from inducing laminB1 protein (Figure 7C). NOTE: The same tubulin control for Figure 5C is used here again in Figure 7C. Both the p21 and LMNB1 (in Figure 5C) were detected on the same gel. They were spearated to optimize narration and flow. Loss of CEBPγ did not impact Snail1’s ability to upregulate SESN2, BAX, BBC3 (PUMA), SOD3, and PMAIP1 (NOXA) mRNA (Figure S1). These results strongly suggest that without CEBPγ, Snail1 may push PCa cells toward irreversible senescence independent of stress-response or apoptotic priming.

## 4. Discussion

**Figure 8:**
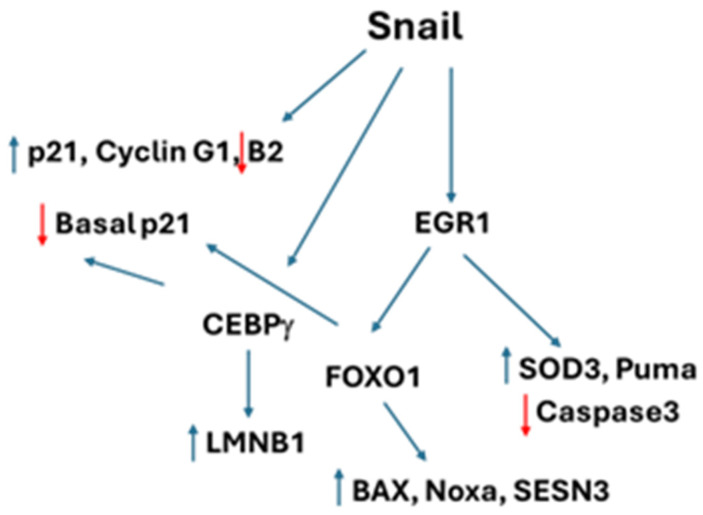
Working model for Snail1 control of cell proliferation, apoptosis, and senescence. Snail1 directly controls expression of p21, cyclins B2 and G1 through promoter binding. Snail1 directly binds to and induces EGR1 to independently control apoptosis/cell stress and through FOXO1 to control additional cell stress/apoptosis molecules. Snail1 directly binds to and induces CEPBγ to prevent senescence and limit basal p21 levels. FOXO1 can also independently control basal p21 levels. Red arrows indicate suppression, blue arrows indicate induction.

By using an inducible expression system that avoids supraphysiologic Snail1 levels, our work refines prior models of Snail1 function and reveals a role for Snail1 in dynamic pathways involving cell proliferation, senescence avoidance, and apoptotic priming, without phenotypic apoptotic execution (Figure 8). Bioinformatics inference and perturbation experiments further indicate that Snail1 engages multiple transcriptional regulators, including FOXO1, EGR1, and CEBPγ, in a context-dependent and non-linear manner. While EGR1 is required for Snail1-dependent induction of FOXO1, neither EGR1, FOXO1, nor CEBPγ are necessary for Snail1-mediated p21 transcription, indicating that p21 induction arises through a distinct arm of the Snail1 program. Evidence that Snail1 binds directly to the p21 (*CDKN1*) gene suggests a direct mechanism. At the protein level, FOXO1 and CEBPγ influence baseline p21 abundance, suggesting that Snail1-driven transcriptional outputs are superimposed upon protein-level setpoints that shape functional outcomes.

A central finding of this work is the role of Snail1 in controlling stress, apoptosis, and senescence. We found that Snail1 induces stress responses and apoptotic programmers through the EGR1/FOXO1 transcriptional axis. However, the EGR1/FOXO1 regulation of caspase 3 expression seems to prevent the induction of apoptosis. In addition, we linked Snail1 to the maintenance of proliferative competence and perseverance of nuclear integrity through its control of LaminB1 [30,32]. The ability of Snail1 to increase LaminB1 protein abundance is dependent on CEBPγ, independently of EGR1/FOXO1. Importantly, this model does not require Snail1 to actively promote senescence; instead, senescence-associated features may emerge when buffering mechanisms such as CEBPγ-dependent LaminB1 maintenance are compromised.

Overall, these observations support a model in which Snail1 imposes a low-proliferative cellular state while simultaneously engaging protective mechanisms that prevent self-induced apoptosis and loss of nuclear integrity. Our findings reposition Snail1 as a regulator of proliferative state that operates through distributed transcriptional, post-transcriptional, and nuclear programs rather than as a unidirectional driver.

These findings also highlight the importance of carefully validating and testing bioinformatic-driven models. Despite our VIPER/DOROTHEA analysis showing probable Snail1-dependent regulatory nodes between FOXO1/p21/cyclin B2 and CEBPγ/p21 [26,56], these pathways were not evident in the experimental data. From the ChIP-Seq data, Snail1’s occupancy at the promoters of direct cell cycle genes suggests direct regulatory action without relying on intermediary actors like ERG1, FOXO1, or CEBPγ. In addition, the known non-transcriptional suppression of Cdk2/4 activity through CEBPαβγ may also impact cell proliferation [27].

In this study, we attempted to frame Snail1 status as a dynamically changing continuum, not a static property, as a mechanism for the initiation of prostate cancer metastasis. We identified clearly distinct mechanisms in the AR+ C4-2B and 22Rv1 cells versus the AR-negative DU145 cells. We specifically chose to focus on the AR+ cells since this is the primary state of prostate cancer cells in the primary tumor that must somehow acquire a signal to initiate metastasis. The status of androgen receptor (AR), its expression versus its activity, could help better define early metastatic events. While prostate cancers express both Snail1 and AR, AR activity is dominant and overrides Snail1 activity [14]. Thus, for AR+ cells to metastasis via a Snail1-dependent mechanism may necessitate a transient decrease in AR activity. What might cause this AR activity drop needs to be determined, because the use of ADT therapeutically is known to reduce AR activity and could actually promote metastasis [14].

These studies open the door to beginning an analysis of these pathways in human tissues. This will not be a simple study, in that it will be necessary to assess both the nuclear-specific expression of Snail1 (i.e., active) and active downstream targets, as well as the active state of AR all within the same cell. Moreover, if it is this type of cell that is metastatic, it will likely represent only a small fraction of the total cells in the tumor sample. One would also suspect such cells would be more prevalent in higher Gleason Grade tumors. One might also consider whether the same pathways active in AR+ tumors would be the same or different in NE or non-AR expressing tumors. Indeed, our results with DU145 cells suggest there are likely differences depending on the tumor subtype.

Snail1 expression in human prostate cancer is prevalent in clinical PCa [5,14]. In such settings, Snail1-associated growth suppression may contribute to tumor persistence by supporting viable, low-proliferative states that remain capable of re-entering the cell cycle upon additional input. From a therapeutic perspective, targeting Snail1-dependent senescence gatekeepers—rather than Snail1 itself—could shift tumor cells toward deeper, less reversible growth-limited states, potentially improving durability of clinical responses. A secondary strategy has many caveats but aligns with existing translational goals: make cancer cells more sensitive to chemotherapy. Targeting the Snail1-p21 axis may rescue PCa cells from proliferation inhibition. Proliferative cells are intricately sensitive to chemotherapy. Because p21 is a master regulator of many processes, targeting p21 can be quite harmful to the patient. We have shown that Snail1 occupies the p21/CDKN1A promoter, concurrent with p21 upregulation. Identifying targetable Snail1 cofactors may confer cancer specific therapy.

## 5. Conclusions

We demonstrated that Snail1 acts as dynamic regulator that enforces a low-proliferative state while activating protective programs that avert apoptotic execu-tion and preserve nuclear integrity. Snail1 primes stress/apoptosis via an EGR1→FOXO1 axis and independently maintains LaminB1 through CEBPγ; senescence-like features likely emerge when these buffering arms fail. p21 is a distinct Snail1 arm, consistent with direct Snail1 binding, while FOXO1/CEBPγ tune baseline p21 protein setpoints rather than being required for Snail1-driven p21 transcription. Clinically, fluctuating Snail1 under anti-AR pressure may stabilize reversible low-cycling tumor states, motivating therapeutic targeting of Snail1-dependent gatekeepers rather than Snail1 itself.

## Data Availability

The original transcriptomics and ChIP data presented in the study are openly available in Geo DataSets at https://www.ncbi.nlm.nih.gov/geo/query/acc.cgi?acc=GSE318340 (accessed on 28 January 2026) and https://www.ncbi.nlm.nih.gov/geo/query/acc.cgi?acc=GSE318342 (accessed on 28 January 2026).

## Supplementary Materials

The following supporting information can be downloaded at: https://www.mdpi.com/article/10.3390/cancers18030510/s1, Table S1: siRNA Sequences; Table S2: Antibodies; Table S3: qPCR Primer sequences; Table S4: RNA-Seq Hits List; Table S5: ChIP-Seq annotated peaks C4-2B; Table S6: ChIP-Seq annotated peaks DU145; Figure S1: Loss of CEBPγ does not impact Snail1’s ability to upregulate p21, SESN2, BAX, BBC3 SOD3 and PMAIP1, and downregulate Cyclin A2 and B2. Figure S2: Raw immunoblot compilation.
