# Supplementary material for: Snail1 Induced Suppression of Proliferation via EGR1, FOXO1, and CEPBγ Creates a Vulnerability for Targeting Apoptotic and Cellular Senescence Pathways
Source: Cancers (Basel). 2026 Feb 4;18(3):510. doi: 10.3390/cancers18030510 (PMC12896619; doi:10.3390/cancers18030510)
Supplement: Supplementary file 1 [file cancers-18-00510-s001.zip › cancers-4104025-supplementary-1.pdf]

Jack Tran [1], Samyukta Sundaram [2], Sukirti Shivpuri [2], Hunain Khawaja [1] and Cynthia Miranti [1,2,*]

Supplemental Data

Materials and methods

Table S1. siRNA sequences

siControl pool sequence 1	UGGUUUACAUGUCGACUAA
siControl pool sequence 2	UGGUUUACAUGUUGUGUGA
siControl pool sequence 3	UGGUUUACAUGUUUUCUGA
siControl pool sequence 4	UGGUUUACAUGUUUUCCUA
siEGR1 pool sequence 1	GAUGAACGCAAGAGGCAUA
siEGR1 pool sequence 2	CGACAGCAGUCCCAUUUAC
siEGR1 pool sequence 3	GGACAUGACAGCAACCUUU
siEGR1 pool sequence 4	GACCUGAAGGCCCUCAAUA
siFOXO1 pool sequence 1	GCGCUUAGACUGUGACAUG
siFOXO1 pool sequence 2	GAGGUAUGAGUCAGUAUAA
siFOXO1 pool sequence 3	UGACUUGGAUGGCAUGUUC
siFOXO1 pool sequence 4	GGACAACAACAGUAAAUUU
siCEBPgamma sequence 1	UCGAAACAGUGACGAGUAU
siCEBPgamma sequence 2	GAACGGAAUUAGUGUUAUC
siCEBPgamma sequence 3	GGAAUUAAGUGUACUCAAA
siCEBPgamma sequence 4	GACAGCAGAUGGCGACAAU

Table S2. Antibodies

Targets	Sources	catalog #	Host
Snail1	Cell Signaling Technologies	C15D3	Rabbit
a-Tubulin	Thermofisher	DM1A	Mouse
p21	Cell Signaling Technologies	2947S	Rabbit
FOXO1	Cell Signaling Technologies	2880T	Rabbit
EGR1	Cell Signaling Technologies	15F7	Rabbit
EpCAM	Cell Signaling Technologies	VU1D9	Mouse
CEBPgamma	Abnova	10581-472	Rabbit
Caspase3	Cell Signaling Technologies	8G10	Rabbit
LMNB1	Cell Signaling Technologies	13435T	Rabbit
Anti-mouse igg	Cell Signaling Technologies	91196	Goat
Anti-Rabbit igg	Cell Signaling Technologies	7074	Goat

Table S3. qPCR primer sequences

Target	Sequence 5'-3'
Snail1 Forward	GCTGCAGGACTCTAATCCAGA
Snail1 Reverse	ATCTCCGGAGGTGGGATG
ERG1 Forward	GAGAAGGTGCTGGTGGAGAC
ERG1 Reverse	CAAGGTGTTGCCACTGTTGG
FOXO1 Forward	ACCTGTACAAGTGCCTCTGC
FOXO1 Reverse	CTTGGGAGCTTCTCCTGGTG
CDKN1A Forward	TCTTGTACCCTTGTGCCTCG
CDKN1A Reverse	ATCTGTCATGCTGGTCTGCC
CCNA2 Forward	ATTGCTGGAGCTGCCTTTCA
CCNA2 Reverse	CATGCTGTGGTGCTTTGAGG
CCNB2 Forward	GGCTCCAAAGGGTCCTTCTC
CCNB2 Reverse	TTGCAGAGCAAGGCATCAGA
CEBP-gamma Forward	ATCGCAGCAAAACAGCACTC
CEBP-gamma Reverse	TGAGGAACCTGCTGTAAGCC
RPL4 Forward	AGGCCAGGAATCACAAGCTC
RPL4 Reverse	TGTCGGAGTACAGCAAGCAG
SESN2 Forward	GCCACTCAGAGAAGGTCCAC
SESN2 Reverse	GAGTCAGGTCATGTAGCGGG
BAX Forward	GCTTCAGGGTTTCATCCAGG
BAX Reverse	CAGCTTCTTGGTTGGACGC
BBC3 Forward	CAGGAAAGCCTGTTGTGCTG
BBC3 Reverse	AAGGAGCACCGAGAGGAGAG
PMAIP1 Forward	GAGGTTCCCGGGCTCTGTAG
PMAIP1 Reverse	CACTCGACTTCCAGCTCTGCT
SOD3 Forward	TCCCTATACCGAGACCCACC
SOD3 Reverse	GGCCTTCAGACCTACTGAGTG

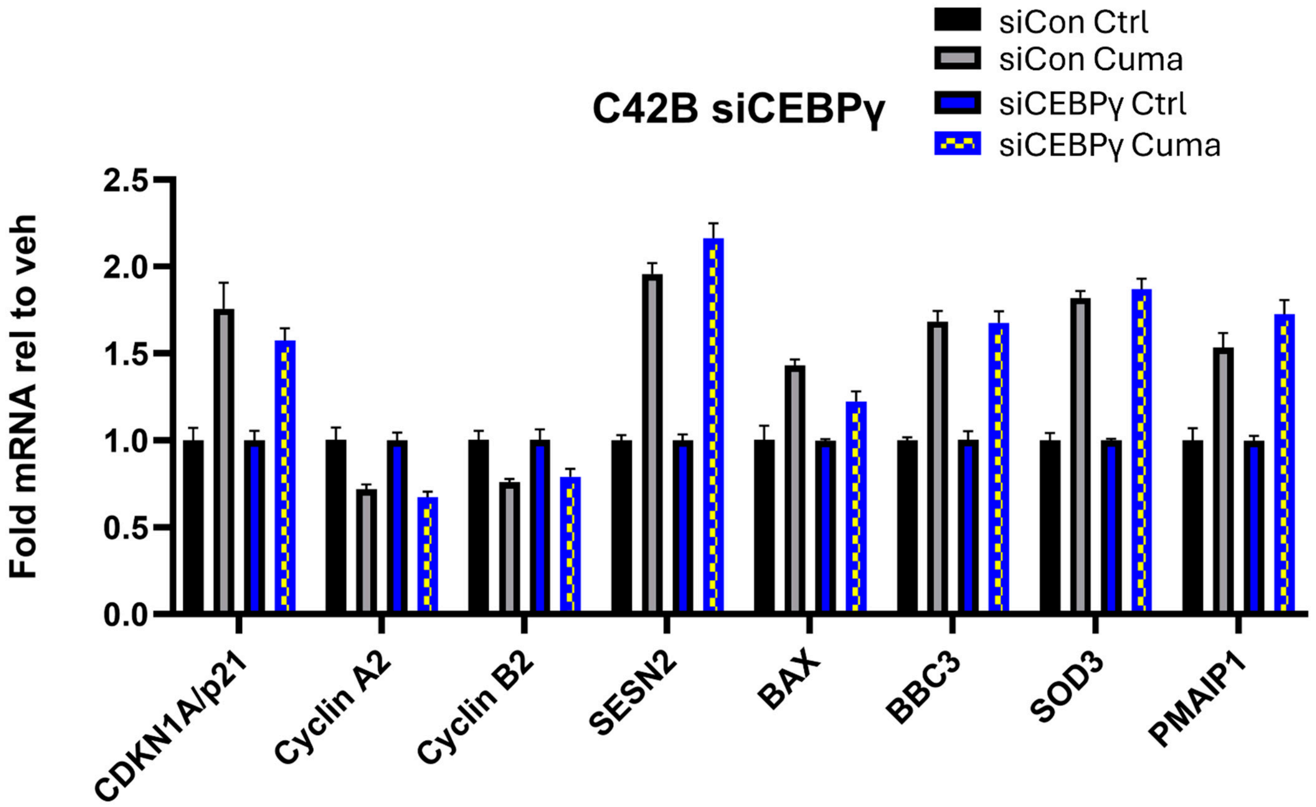

Figure S1: Loss of CEBPγ does not impact Snail1's ability to upregulate p21, SESN2, BAX, BBC3 SOD3 and PMAIP1, and downregulate Cyclin A2 and B2. C4-2B cells were treated with 4.5ug/ml Cumate (Cuma) or H_2O (Ctrl) for a total of 6 days. In the last 48h, cells were treated with siCEBPγ or siControl. Fold change analysis normalized to Ctrl groups. SESN2 and SOD3 are stress-response markers. BAX, BBC3 (PUMA) and PMAIP1 (NOXA) are apoptotic programming markers. Color code: siCon(black/gray), siCEBPγ (blue)

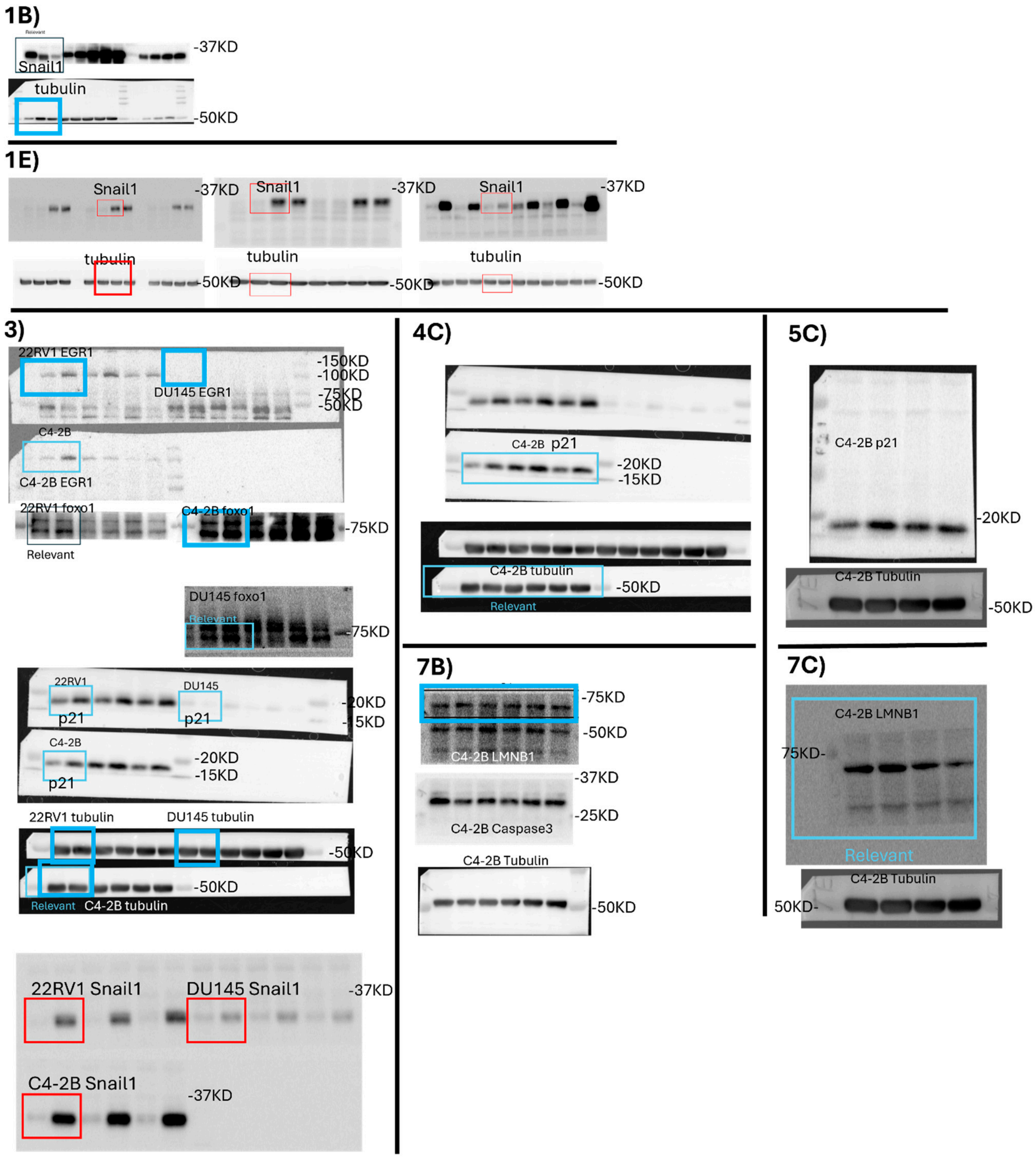

Figure S2: Raw immunoblot compilation. Labeled sections correspond to figure labels on main manuscript. Wherever applicable, PVDF membranes are cut in strips for the purpose of detecting specific targets at specific sizes, as noted with KD notation. Example: **1B)** Snail1 is detected below the 50KD mark; whereas Tubulin is detected above the 50KD mark. Thus the blot membrane was cut right below 50KD for Snail1 detection, and the section above the 50KD was used to detect tubulin. As noted for Figure 5C and 7C in manuscript, loading control tubulin is used for both figures, because p21 and LMNB1 were ran on the same gel as the tubulin control. Each gene was presented in separate figures for the purpose of narrative ordering and logics. **3)** p21 levels in DU145 is generally low. The raw image presented is meant to illustrate data fidelity, not readability. Readers/reviewers are encouraged to adjust light saturation levels to get the DU145's p21 signal to show up, for the purpose of readability.
